# Testing Navigation in Real Space: Contributions to Understanding the Physiology and Pathology of Human Navigation Control

**DOI:** 10.3389/fncir.2020.00006

**Published:** 2020-03-06

**Authors:** Florian Schöberl, Andreas Zwergal, Thomas Brandt

**Affiliations:** ^1^Department of Neurology, University Hospital, Ludwig Maximilian University (LMU) of Munich, Munich, Germany; ^2^German Center for Vertigo and Balance Disorders, DSGZ, LMU Munich, Munich, Germany; ^3^Clinical Neurosciences, LMU Munich, Munich, Germany

**Keywords:** visual exploration, landmarks, navigation, egocentric and allocentric navigation, brain imaging, spatial disorientation, hippocampus

## Abstract

Successful navigation relies on the flexible and appropriate use of metric representations of space or topological knowledge of the environment. Spatial dimensions (2D vs. 3D), spatial scales (vista-scale vs. large-scale environments) and the abundance of visual landmarks critically affect navigation performance and behavior in healthy human subjects. Virtual reality (VR)-based navigation paradigms in stationary position have given insight into the major navigational strategies, namely egocentric (body-centered) and allocentric (world-centered), and the cerebral control of navigation. However, VR approaches are biased towards optic flow and visual landmark processing. This major limitation can be overcome to some extent by increasingly immersive and realistic VR set-ups (including large-screen projections, eye tracking and use of head-mounted camera systems). However, the highly immersive VR settings are difficult to apply particularly to older subjects and patients with neurological disorders because of cybersickness and difficulties with learning and conducting the tasks. Therefore, a need for the development of novel spatial tasks in real space exists, which allows a synchronous analysis of navigational behavior, strategy, visual explorations and navigation-induced brain activation patterns. This review summarizes recent findings from real space navigation studies in healthy subjects and patients with different cognitive and sensory neurological disorders. Advantages and limitations of real space navigation testing and different VR-based navigation paradigms are discussed in view of potential future applications in clinical neurology.

## Introduction

In the last decades, we have gained fundamental insight into human navigation control from case studies in patients with circumscribed cerebral lesions and functional MRI (fMRI) studies using navigation tasks in well-controlled and distinct virtual reality (VR) settings (Maguire et al., 1998, 2000, 2003, 2006; Astur et al., 2002; Boccia et al., 2014; McCormick et al., 2017, 2018). However, performing navigation tasks in VR has obvious methodological limitations. The need for a stationary body position (e.g., in the MRI scanner) may result in a bias towards optic flow and visual landmark processing. In contrast, real space navigation relies on simultaneous processing of visual, vestibular, proprioceptive and motor-efference signals, as well as on their integration and weighting for online spatial updating of one’s position in space (Loomis et al., 1993; Waller et al., 2003; Ruddle and Lessels, 2006; Waller and Lippa, 2007; Ruddle et al., 2011; Chrastil and Warren, 2012, 2013; Bates and Wolbers, 2014; Ekstrom and Isham, 2017; Diersch and Wolbers, 2019). Input from the otoliths contributes only to 2-dimensional (2D) egocentric navigation in stationary subjects, while input from otoliths and semicircular canals is used to guide 3-dimensional (3D) allocentric navigation in mobile subjects (Brandt and Dieterich, 2016). Recent technological developments have improved the capability to include multisensory input in VR. Examples are the highly immersive VR settings displayed in head-mounted goggles and hybrid approaches using treadmill locomotion in immersive VR (Diersch and Wolbers, 2019; Huffman and Ekstrom, 2019; Starrett et al., 2019) or *post hoc* imagination and simulation of real space environments (Hirshhorn et al., 2012; Howard et al., 2014; Brunec et al., 2018; Patai et al., 2019). Under these conditions, however, simultaneous measurements of brain activation (by fMRI) are a particular challenge. Furthermore, the application of such highly immersive and hybrid VR approaches may be limited by cybersickness and deficits in handling due to decreased embodiment, particularly in elderly subjects and patients with neurological disorders (Costello and Bloesch, 2017; Diersch and Wolbers, 2019). Consequently, novel navigation paradigms in real space have been developed, which allow for synchronous analysis of navigation performance, visual explorations and brain activation in different natural environments. The aim of this review article is: (1) to summarize recent experiences with the application of real space navigation tasks in healthy subjects and patients with cognitive and sensory deficits; and (2) to discuss the advantages, limitations and potential future clinical applications of real space navigation tests compared to currently available VR based set-ups.

## Navigation Testing in Healthy Subjects

### 2D Navigation: Behavior, Cerebral Networks and Current Concepts

Human navigational behavior critically depends on the surrounding environment, specifically the spatial dimension, scale and the abundance of visual cues suitable as landmarks. Most of the human real-life navigation takes place in 2D environments within four major vectors of movement (i.e., forward/backward, right/left). Spatial scale can vary greatly from vista-space (e.g., navigation within a room) to large-scale environments (e.g., navigation in a city or landscape).

In the late 1990s, first studies combined fMRI techniques with stationary desktop VR settings to study human 2D spatial navigation control. These studies revealed a distributed cerebral navigation network in humans consisting of frontal lobe regions, mesiotemporal regions (hippocampus and parahippocampal cortex), parietal lobe regions [posterior parietal cortex (PPC) and retrosplenial cortex (RSC)], as well as subcortical regions (basal ganglia and thalamus; Ghaem et al., 1997; Epstein and Kanwisher, 1998; Maguire et al., 1998; Epstein et al., 1999, 2017; Grön et al., 2000; Burgess et al., 2002; Hartley et al., 2003; Spiers and Maguire, 2006; Ekstrom et al., 2014; Epstein and Vass, 2014; Ekstrom, 2015; Ekstrom and Isham, 2017). fMRI approaches in more complex VR environments pointed towards a particular and navigation-specific role of the hippocampal formation, the RSC and PPC (Berthoz, 1997; Hartley et al., 2003; Ohnishi et al., 2006; Spiers and Maguire, 2006; Wolbers et al., 2007, 2008; Brown and Stern, 2014; Howard et al., 2014; Marchette et al., 2014, 2017; Spiers and Gilbert, 2015; Ekstrom and Isham, 2017; Epstein et al., 2017; Vass and Epstein, 2017; Huffman and Ekstrom, 2019; Patai et al., 2019). VR set-ups presenting cityscapes and landscapes, as well as visual scenarios, induced strong activation in the parahippocampus, RSC and PPC thus confirming their particular role in visual cue and scene processing (Epstein and Kanwisher, 1998; Spiers and Maguire, 2006). More advanced task designs in VR and progress of fMRI techniques allowed further differentiation of visual scene and cue processing within the human brain. The parahippocampal place area (PPA) located directly posterior to the parahippocampus is important for the recognition of visual scenes and particularly the identification and selection of visual landmarks within a visual scenario (Epstein et al., 2005). The occipital place area (OPA) located in the dorsal occipital lobe next to the transverse occipital sulcus is mandatory for the recognition of the spatial geometry and particularly boundaries of a presented visual scene, as well as the local elements (Dilks et al., 2013; Kamps et al., 2016). The RSC located dorsally to the splenium of the corpus callosum within the medial parietal cortex uses the selected landmarks to determine the current location in space, as well as heading/facing direction in space (Epstein and Vass, 2014; Vass and Epstein, 2017). Thus, VR-based scene processing and navigation tasks are well suited to study the brain networks and principles of human visual cue and scene processing when combined with advanced fMRI techniques.

**Figure 1:**
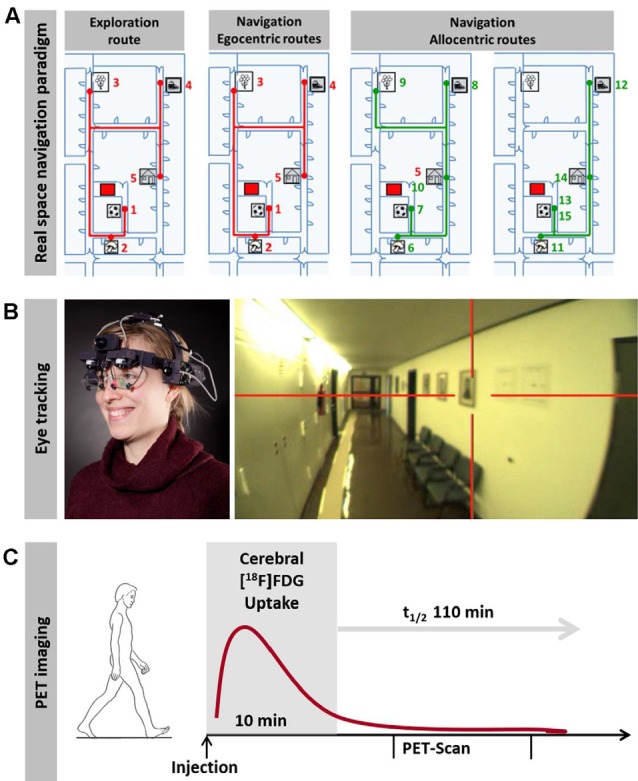
Horizontal real space navigation paradigm. **(A)** real space navigation is tested in a complex and unfamiliar spatial environment, where items are placed as target points. These items are shown to the subjects first on an investigator-guided walk (exploration). Afterward, subjects have to find the items in a pseudo-randomized order over the next 10 min (navigation). In the first part of the navigation paradigm, routes, which are identical to the previous exploration route (so-called egocentric routes, red lines), are tested; in the second part, the order of target items is changed in a way that requires the planning of new routes (so-called allocentric routes, green lines). **(B)** Subjects wear a gaze-monitoring head camera throughout the experiment to allow *post hoc* analysis of their visual exploration. **(C)** [^18^F]FDG is injected at the start of the 10-min navigation phase. After the end of navigation testing subjects rest in a supine position for 20 min and image acquisition starts 30 min after tracer administration. This paradigm can be used to depict navigation-induced brain activations because the cerebral glucose utilization is weighted to the 10 min following [^18^F]FDG injection and is integrative due to intracellular trapping of the tracer (adapted from Irving et al., 2018b; permission from the journal obtained).

Complementary to VR experiments, real space navigation paradigms have been developed, which combine synchronous measurements of navigation behavior, visual exploration and navigation-induced brain activation (using [^18^F]fluorodeoxyglucose ([^18^F]FDG)-PET; Zwergal et al., 2016; Irving et al., 2018b; Figure 1). Real space navigation paradigms are prone to study natural visual exploration behavior (visual fixations, saccadic eye movements) by eye-tracking (Irving et al., 2018b). Furthermore, navigation-induced brain activations can be captured by [^18^F]FDG-PET, because more than 90% of [^18^F]FDG accumulates in neurons activated by real navigation within 10 min. Recent studies have shown that 2D real space navigation activated a cerebral network consisting of the hippocampus, posterior temporal, RSC, parieto-occipital and frontal cortex, as well as the pontine brainstem centers for horizontal eye movement control (Zwergal et al., 2016). Cerebral control of real space navigation shows considerable overlap with regions reported in VR experiments. However, there are also differences in navigation strategies and brain activations in VR and real space:

(1) Brain regions important for processing of novel visual scenes such as the PPA and OPA are not active during real space navigation experiments. The most likely explanation is that transient brain activations during a confrontation with a novel spatial environment are not reflected by PET due to low temporal resolution (Zwergal et al., 2016). Furthermore, most real space navigation experiments are preceded by an exploration of the environment. Consequently, the space during navigation is not novel anymore.
(2) Visual cue-based and location-based strategies are used to a different extent in VR and real space environments (Bohbot et al., 2012; Chersi and Burgess, 2015; Ekstrom et al., 2017; Starrett and Ekstrom, 2018). Continuous integration of vestibular and proprioceptive afferent inputs during self-motion enables online updating of our current position in space—a way to navigate successfully even in the absence of landmark processing. The technical term for this navigational strategy is path integration and the underlying computations mainly take place in the PPC and to some extent also in the basal ganglia (Packard and McGaugh, 1992; Packard and Knowlton, 2002; Iaria et al., 2003; Yin and Knowlton, 2006; Hwang and Andersen, 2009; Pennartz et al., 2011; Hwang et al., 2014). The PPC seems to be a hub region for the estimation of distances and heading/body directions (Guariglia et al., 2005; Weniger et al., 2009, 2011; Ciaramelli et al., 2010), while the basal ganglia are indispensable for stimulus-response learning, i.e., connecting a distinct sensory cue to a specific action (Packard and Knowlton, 2002; Iaria et al., 2003). Location-based strategies tend to be underrepresented in VR based tasks due to the lack of idiothetic, i.e., vestibular and proprioceptive input. We know from research in rodents, that passive movement in VR induces other vestibular signals than active movement in real environments, which in consequence has also effects on upstream cell systems important for spatial navigation (i.e., head direction cells, grid cells and place cells; Stackman and Taube, 1997; Stackman et al., 2002; Russell et al., 2003a,b; Taube et al., 2013; Yoder and Taube, 2014). Accordingly, in humans head movements in the horizontal plane are mandatory for regular processing of the head direction system and upstream cell systems for spatial navigation (Frissen et al., 2011). In fact, numerous behavioral studies suggest that body-based cues enhance spatial representations in humans by aiding encoding and retrieval of spatial information (Waller et al., 2003; Ruddle and Lessels, 2006; Ruddle et al., 2011; Chrastil and Warren, 2013). Novel VR technologies such as head-mounted VR systems try to allow for linear and rotational movements by high immersion to the virtual environment. However, this approach excludes the simultaneous capturing of brain activations by fMRI (Diersch and Wolbers, 2019). Furthermore, despite ongoing improvements of VR technology, vestibular and visual inputs are often still not matching perfectly in these VR designs thus leading to manifest cybersickness or at least vestibular discomfort and dizziness particularly in the older participants (Diersch and Wolbers, 2019). Real space navigation paradigms are still the gold standard for the exact assessment of the role of vestibular and proprioceptive processing in humans, at least when using a multimodal approach with eye tracking for *post hoc* analysis of eye and head movements and simultaneous measurement of brain activations by [^18^F]FDG-PET (Zwergal et al., 2016; Irving et al., 2018b). Indeed, during real space navigation, there is significantly higher recruitment of vestibular brain regions in the brainstem and cerebellum, as well as cortical regions important for vestibular and multisensory processing such as the cingulate and insular cortex (Zwergal et al., 2016; Irving et al., 2018b). During the last years, the novel “hybrid-approaches” has come up, which have the potential to further overcome existing limitations of dedicated VR technologies. One strategy combines the simultaneous application of immersive head-mounted VR systems, which allows for free head movements, with continuous walking on a treadmill to simulate locomotion within the VR (Huffman and Ekstrom, 2019; Starrett et al., 2019). Despite the multisensory nature of these approaches, they still differ significantly from the navigational situations in everyday life, because there is no one by one translation of step size to the VR. The training sessions before the trial sessions are very extensive, which is definitely not the case in most real space navigation situations. Application in older people and particularly patient groups with cognitive decline or sensory deficits is still difficult due to technical challenges and the high risk of cybersickness. Previous studies have found no differences in spatial learning of novel environments with addicting treadmill walking to immersive head-mounted VR, which suggests a modality-independent network for retrieval of spatial layout information in humans and questions the relevance of hybrid-approaches (Wolbers et al., 2011). As imaging of brain activation is concerned, fMRI can be applied only *post hoc* by memorizing or simulating navigational situations within the VR. An alternative approach for studying spatial orientation in fMRI is the *post hoc* imagination of navigation through recently learned real space layouts (e.g., London Soho’s streets; Howard et al., 2014; Javadi et al., 2017; Brunec et al., 2018; Patai et al., 2019). The imagination of real tasks in fMRI has been successfully established for the investigation of body movements, standing and locomotion under various conditions (Jahn and Zwergal, 2010; Zwergal et al., 2012). A direct PET/fMRI comparison of real space and imagined locomotion has shown similarity in activated brain networks (e.g., cerebellar activations), but also differences as a function of the task (e.g., primary motor cortex activation in real locomotion vs. prefrontal-basal ganglia activation in imagined locomotion; la Fougère et al., 2010). In navigation research, fMRI imagination paradigms gave valuable insights into the differential contributions of the anterior hippocampus and entorhinal cortex (i.e., gist-like, scheme-like global spatial representations and Euclidean distance), the posterior parts of the hippocampus (i.e., detail-rich spatial representations) and the medial prefrontal cortex (PFC; i.e., decision making, detours). These paradigms helped to support the Multiple Trace Theory (MTT) respectively the Trace Transformation Theory (TTT) as a concept of memory generation and consolidation because they allowed comparing brain activations in familiar environments (e.g., RSC) to those in novel environments (e.g., posterior hippocampus; Nadel and Moscovitch, 1997; Nadel et al., 2000; Patai et al., 2019). The task-inherent need for decision-making at way crossings and the reliably high success rates in correct wayfinding underline that imagination protocols are in principle valid and reliable. However, these approaches are not real space navigation in a narrower sense, as the trial sessions are done while lying supine in an fMRI-scanner and mentalizing the before learned real space environments. An additional problem may be the ability for imagination, which varies greatly between subjects and can be bearly controlled.
(3) The flexible interplay of landmark-based and location-based navigation is fundamental for the creation of metric map-like representations of space. Such a cognitive map seems to be the scaffolding for navigating most efficiently and accurately by using short cuts and being able to react flexibly to unforeseen obstacles and changes in routinely used routes (Epstein et al., 2017). It has been shown that a larger hippocampal volume is associated with superior performance in learning allocentric spatial relationships between buildings on a college campus and allocentric topography of an artificial landscape (Hartley and Harlow, 2012; Schinazi et al., 2013). fMRI techniques such as fMRI adaptation in the last years brought evidence that a spatial map like code exists in the human hippocampus (Hassabis et al., 2009; Doeller et al., 2010; Morgan et al., 2011; Nielson et al., 2015). However, in many VR based fMRI studies, hippocampal activations were not found. It is still a matter of debate, whether the hippocampus or rather the RSC is the main hub for human spatial navigation due to controversial findings from previous fMRI studies (Epstein and Vass, 2014; Ekstrom and Isham, 2017). PET-based measurement of cerebral glucose metabolism while absolving a real space navigation task confirmed the prominent role of simultaneous hippocampal as well as RSC activations for human navigation, thus indicating that these two brain regions are the critical hubs for human navigation in naturalistic and novel environments (Zwergal et al., 2016; Irving et al., 2018b). Furthermore, the results from real space navigation studies favor the view that there indeed is no hierarchical order between these two hubs.
(4) In real space navigation experiments the frontal lobe is regularly activated, which is not the case in many VR tasks. Only a few fMRI studies in VR have shown frontal lobe activations, mostly in the PFC. These studies used tasks that explicitly examined decision making at way crossings with multiple-choice or during detour planning (Kaplan et al., 2017). Real space navigation in novel environments always requires planning processes for routes and decision making at way crossings, which leads to continuous frontal lobe activations. Therefore, it has to be seen as bias and weakness of several VR settings that frontal lobe activations are underrepresented.

### 3D Navigation: Behavior, Cerebral Networks and Current Concepts

Everyday life navigation mostly takes place in the horizontal plane. However, in some situations, such as finding the correct way in multi-floor buildings, humans also need to deal with an additional third dimension of space (3D) and to orient themselves correctly in the vertical plane (Thibault et al., 2013; Brandt et al., 2017). Previous studies in different animal species over the last decades revealed an anisotropy of performance and cellular activations in favor of the prevailing plane in ground-based species, such as dogs and rats, as well as flying species, such as bats and hummingbirds (Burt de Perera and Holbrook, 2012; Burt de Perera et al., 2013; Jeffery et al., 2013; Brandt and Dieterich, 2013; Flores-Abreu et al., 2014).

**Figure 2:**
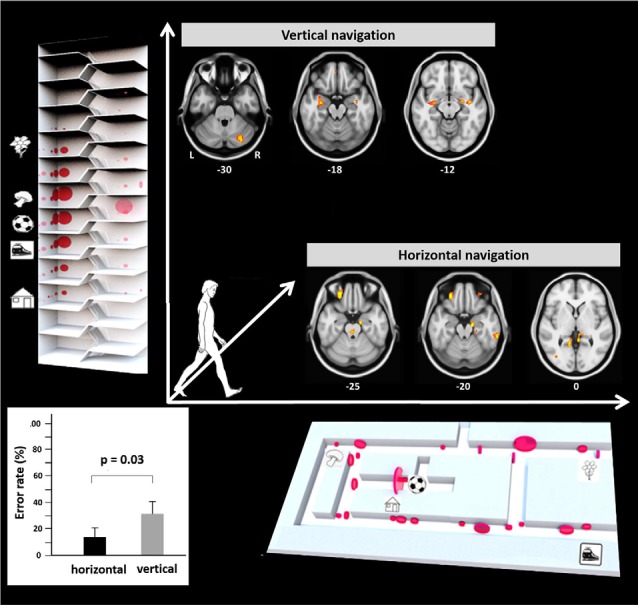
Earth-horizontal vs. earth-vertical real space navigation in healthy subjects. The individual error rate in finding the right items was significantly higher in the vertical than in the horizontal navigation paradigm (bottom left). In the horizontal navigation paradigm selection and recall of landmarks were orientated to strategic waypoints along the path like crossings and prominent items (bottom right). This strategy reflects the importance of landmark-guided navigation in the horizontal plane. In contrast, during vertical navigation landmark fixations were distributed more evenly along the path (top left). This strategy may indicate that vertical navigation is less reliant on visual landmarks. The most frequent fixation targets are indicated as red circles with the diameter being proportional to the mean number of fixations. During horizontal navigation, regional cerebral glucose metabolism was relatively increased in the right anterior hippocampus, bilateral retrosplenial cortex (RSC), and the pontine brainstem tegmentum. The glucose uptake in the eye muscles was higher in horizontal navigation, indicating increased exploration. During vertical navigation regional cerebral glucose metabolism was relatively increased in the anterior hippocampus, insula, and cerebellum bilaterally. Significance level *p* < 0.005, L, left; R, right side, level of the section are marked by MNI-z-coordinates (adapted from Zwergal et al., 2016; permission from the journal obtained).

Recently, a study in humans for the first time investigated performance, visual exploration, and brain activation during real space navigation in either the earth-horizontal or earth-vertical plane (Zwergal et al., 2016; Figure 2). Similar to other ground-based species, an anisotropy of performance in favor of navigation in the horizontal plane was observed in humans (Jeffery et al., 2013; Zwergal et al., 2016). Detailed analysis of behavioral parameters revealed a more prominent role of visual cue processing during horizontal navigation, whereas vertical navigation seemed to rely more on non-visual sensory input such as the vestibular sense (Zwergal et al., 2016). The annotation of visual fixation targets to the spatial environment indicated a weighted distribution to strategic waypoints in the horizontal navigation task compared to a more even distribution in the vertical navigation task (Figure 2). In terms of brain activation, the hippocampus and RSC were activated in both conditions (Figure 2). During horizontal navigation, there was a clear right-sided dominance of the hippocampal activations, whereas during vertical navigation hippocampal activations were bilateral and of similar extent (Zwergal et al., 2016). The higher reliance on visual cues during horizontal navigation was reflected by increased activations of secondary visual cortical areas. During vertical navigation vestibular cortical processing within the insular cortex and anterior cingulate cortex was detected (Zwergal et al., 2016).

In contrast to the results of 3D real space navigation, a recent fMRI study in a virtual 3D lattice structure revealed no difference in performance for the horizontal and vertical plane. Activations of the right hippocampus and RSC were similar for movements along the horizontal and vertical axis (Kim M. et al., 2017; Kim and Maguire, 2019a,b). Differences in results between real space and VR 3D navigation testing are explained mostly by the applied task design. Participants moved in the virtual 3D lattice structure by a keyboard using mainly visual cue processing, while sitting. Given the lack of vestibular and proprioceptive input, missing activations of vestibular and somatosensory brain regions seem plausible. The poorer performance in vertical real space navigation could be due to differences in scale and texture of the environment. The 2D horizontal environment was rich in visual cues and on the border from vista- to large-scale, whereas the 3D vertical environment had less potential landmarks and was definitely large-scale (Zwergal et al., 2016). Another important factor might be that during naturalistic vertical navigation in a staircase, subjects always have conflicting reference frames for visual input (earth-horizontal) and vestibular input (earth-vertical). In contrast, during horizontal 2D navigation, the visual and vestibular reference frames match in the earth-horizontal plane. Given the importance of vestibular inputs for path integration in the vertical plane, a largely visually guided VR-task (like a 3D lattice structure) may not be optimal to depict the subtle difference in 3D navigation performance and cerebral navigation control.

## Navigation Disorders in Humans

Given the complex control of human navigation, it seems self-evident that sensory deficits and dysfunctions of critical hubs of the cerebral navigation network (e.g., the hippocampus, RSC, PPC) lead to spatial navigation deficits in humans. However, no established tests are available in clinical routine to detect spatial orientation deficits in an easy and reliable way. Consequently, there is a pressing need to transfer knowledge from the laboratory setting to the bedside of affected patients. Neurological navigation disorders can be classified based on the underlying pathology in patients with: (1) sensory deficits; (2) acute strategic cerebral lesions; and (3) chronic dysfunction of cerebral navigation networks (mostly due to neurodegeneration).

### Sensory Deficits: Bilateral Vestibular Failure

Research in rodents disclosed how vestibular inputs reach the hippocampal formation *via* different brainstem and thalamic pathways (Smith, 1997; Shinder and Taube, 2010). Accordingly, complete bilateral vestibular deafferentation results in hippocampal cell loss as well as pronounced and permanent deficits in spatial learning tasks in rodents (Smith et al., 2005, 2015; Zheng et al., 2009; Baek et al., 2010). The neurophysiological correlate of this behavioral finding is a pronounced impairment of the head direction cell system as a direct consequence of a missing vestibular input (Yoder and Taube, 2014). Additionally, there is also a dysfunction of the place cells and grid cells in rodents with vestibular deficits (Stackman et al., 2002). Brandt and colleagues were the first to show that complete bilateral vestibular deafferentation in humans resulted in hippocampal volume loss and concomitant deficits of spatial learning in a virtual version of the Morris water maze task, while other cognitive functions, such as memory, executive or visuoconstructive functions, remained unaffected (Brandt et al., 2005). Desktop VR based spatial learning tasks in vista space revealed mild deficits of allocentric place learning and an atrophy of the middle and posterior parts of the hippocampal formation in patients with bilateral vestibular failure (BVF; Schautzer et al., 2003; Kremmyda et al., 2016). A fMRI study in a large-scale virtual environment showed that impaired place learning in patients with incomplete BVF may be compensated by cerebellar-driven sequence-based spatial navigation (Jandl et al., 2015).

All previous VR based studies in vestibular pathologies have the major limitation that they cannot fully account for the role of different sensory inputs for spatial updating as patients sit or lie during the task (Taube et al., 2013). Consequently, spatial navigation testing in patients with sensory deficits should be preferably applied in real space settings, where vestibular signals from the otoliths (linear motion) and semicircular canals (rotatory motion), as well as proprioceptive signals, contribute to the head direction and grid cell function (Taube et al., 2013; Yoder and Taube, 2014; Cullen and Taube, 2017). In a real space navigation paradigm, mild deficits in allocentric route learning were found in patients with BVF compared to healthy and age-matched controls. The allocentric navigational performance was better in patients with residual vestibular function. In contrast, egocentric navigation was well preserved in the BVF patients. When analyzing the navigational behavior, patients with BVF showed characteristic repetitive stops along the way, which allowed for updating of the position in space by landmark fixations. Patients with BVF had less right-sided hippocampal activations and more activation in the bilateral PPAs during navigation (Zwergal et al., 2011). This pattern likely reflects a more landmark-based strategy to compensate for deficient allocentric map-like coding.

### Acute Strategic Cerebral Lesions: Stroke and Transient Global Amnesia

**Figure 3:**
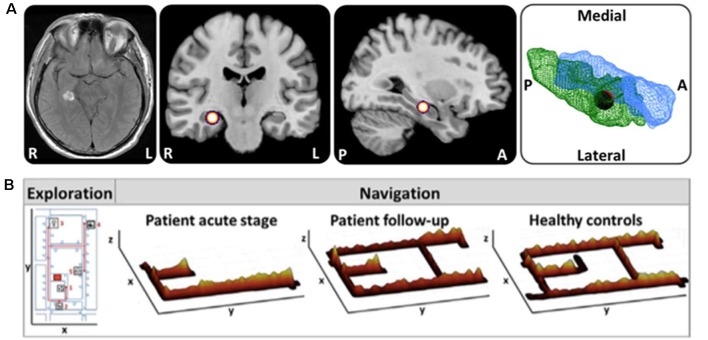
Horizontal navigation in a patient after acute hemorrhage of the right posterior hippocampus. **(A)** Schematic drawing of the exact lesion localization in the right posterior hippocampus and adjacent parahippocampal cortex. **(B)** In the acute stage the patient exhibited severe topographical disorientation with a completely missing cognitive map of the environment. In a follow-up investigation after 4 months, the patient’s navigation performance was completely normalized with an intact cognitive map of the environment. Search paths during navigation are color-coded on a ground map (x, y) as the cumulative time at the location (z). The most frequent fixation targets are indicated as green circles on the ground map with diameter proportional to the cumulative time of fixation (adapted from Irving et al., 2018a; permission from the journal obtained). L, left; R, right.

It has been well documented by single case reports and small case series that acute or subacute lesions in critical brain regions can affect spatial navigation abilities dramatically, leading to severe topographical disorientation (Aguirre et al., 1996; Aguirre and D’Esposito, 1999; Nyffeler et al., 2005; Ritchie et al., 2018). However, a valid and structured classification system and generally accepted taxonomy of spatial navigation deficits are still missing. Recently, Claessen and van der Ham (2017) tried to classify the different clinical forms of topographagnosia based on a retrospective analysis of published lesion cases. Deficits in visual landmark encoding, location, and path processing were rated for this purpose (Claessen and van der Ham, 2017). The major limitation of this retrospective approach was the great heterogeneity in lesion size and localization, as well as time points and methods of spatial navigation testing. For the future, it seems reasonable to tests patients with strategic lesions of the hippocampal formation, RSC and parietal cortex for deficits of ego- and allocentric navigation in a standardized way. Furthermore, it is important to characterize the functional outcomes of patients with distinct lesions affecting the cerebral navigation network. Several case reports showed the great potential for functional compensation in the human spatial navigation network, when only a circumscribed part is affected (Figure 3; Irving et al., 2018a). Although not known exactly, it might be possible that intact subfields of the affected hub or other critical hubs within the cerebral navigation contribute to the compensation of the spatial navigation performance and strategy.

**Figure 4:**
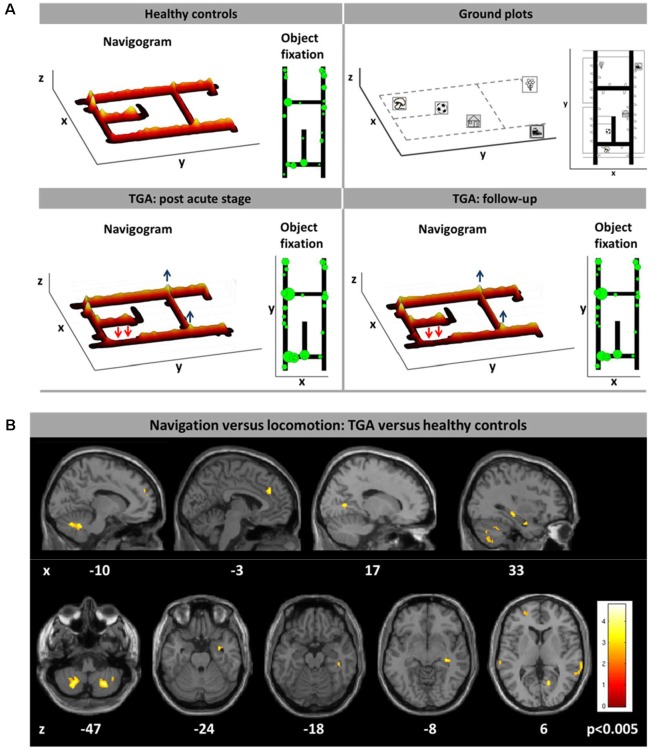
Horizontal real space navigation in patients with transient global amnesia (TGA). **(A)** TGA-patients, as compared to healthy controls, had a different navigation strategy in the post-acute stage (day 3 after symptom onset) with less usage of short cuts (red arrows), a longer duration at strategic way crossings (blue arrows) and more visual fixations along the path, particularly at strategic way crossings. This pattern persisted until follow-up after 3–4 months. Search paths during navigation are color-coded on a ground map (x, y) as the cumulative time at the location (z). The most frequent fixation targets are indicated as green circles on the ground map with diameter proportional to the cumulative time of fixation. **(B)** During navigation in the post-acute stage the regional cerebral glucose metabolism in TGA-patients was increased in the right hippocampus, bilateral posterior parietal, retrosplenial, mesiofrontal cortex, and the cerebellar dentate nucleus, indicating compensatory recruitment of the hippocampal and extrahippocampal navigation network. Significance level *p* < 0.005; the level of sections is given in MNI-coordinates (adapted from Schöberl et al., 2019; permission from the journal obtained).

Besides well-selected lesion patients due to stroke or brain trauma, TGA seems to be a selective hippocampal lesion model in humans. TGA is a disorder of as far unknown etiology manifesting with sudden onset of anterograde and retrograde amnesia lasting for 1–24 h (Bartsch and Deuschl, 2010; Bartsch and Butler, 2013). MRI regularly depicts focal lesions in the lateral CA1 regions of the hippocampus on one or both sides during the acute stage of disease (Bartsch et al., 2006). As a model of intermittent hippocampal dysfunction, it is of particular interest, whether and to what extent spatial learning and navigation are affected in these patients. Bartsch and colleagues have shown that hippocampus-based place learning is critically impaired in patients with TGA in the very acute stage when they still suffer from anterograde amnesia (Bartsch et al., 2010). A recent study in real space confirmed an allocentric navigation deficit in TGA patients in the post-acute stage (3 days after symptom onset), when verbal and figural memory functions had already normalized (Schöberl et al., 2019). Navigation deficits were accompanied by altered visual explorations reflecting a higher reliance on visual landmarks (Schöberl et al., 2019; Figure 4A). PET measurements in the post-acute stage showed increased brain activations of the right hippocampus and bilateral RSC, posterior parietal and mesiofrontal cortex (Schöberl et al., 2019; Figure 4B). These findings can be interpreted as a compensatory upregulation of the human cerebral navigation network including the regions for allocentric spatial representations (i.e., hippocampus and RSC), egocentric spatial representations (i.e., PPC) and for the planning of the applied spatial strategy (i.e., mesiofrontal cortex). Contrary to previous reports, allocentric navigation deficits in real space persisted up to 4 months after symptom onset. Egocentric wayfinding was not affected in the patients at any time point. These real space findings show that strategic bilateral lesions in hippocampal subfields in TGA can induce severe spatial disorientation, even in the presence of a functional extrahippocampal navigation network. Compensation may be less effective than in unilateral hippocampal lesions.

### Neurodegenerative Disorders: Mild Cognitive Impairment and Alzheimer’s Disease

In the clinical setting, spatial disorientation often is the leading symptom in patients with Alzheimer’s disease (AD; Tu and Pai, 2006). This clinical observation could be confirmed by several studies, which tested spatial learning abilities either by VR or in real space in patients with early AD (Pai and Jacobs, 2004; Cushman et al., 2008; Allison et al., 2016). From a neuropathological viewpoint, it is not surprising that spatial navigation is affected early in the course of AD and progresses dramatically over time. Neuropathological changes, such as tau-deposits, synaptic dysfunction, and neuronal loss, manifest very early in critical hubs of the human spatial navigation network such as the mesiotemporal lobe (i.e., hippocampus and entorhinal cortex) and RSC (Braak et al., 1993; Braak and Braak, 1995, 1998; Vlček and Laczó, 2014). Research in the last decades revealed that navigation deficits can be already found in patients with amnestic mild cognitive impairment (aMCI), which is thought to be a pre-stage of impeding dementia (Hort et al., 2007; Laczó et al., 2009, 2010; Lithfous et al., 2013; Vlček and Laczó, 2014; Rusconi et al., 2015; Kim J. W. et al., 2017). Spatial navigation testing, therefore, has great potential for the early diagnosis of aMCI/AD in pre-symptomatic stages (Coughlan et al., 2018). Multiple-domain aMCI patients, i.e., patients with deficits in more than one cognitive subdomain, exhibit deficits in virtual allocentric and egocentric spatial navigation tasks, whereas single-domain aMCI patients still seem to have intact egocentric spatial navigation abilities (Hort et al., 2007). In previous aMCI/AD navigation research, different VR-based or small-scale real space tasks were applied, which at least might bias the transfer to everyday life navigational situations. Only a few studies investigated visual exploration patterns during spatial navigation. Only advanced analysis of eye movements allows for differentiation of deficits in higher-order visual functions, such as the selection of appropriate visual cues, from deficits in visual scene processing or deficits in estimating directions and distances. More advanced VR technologies with a higher immersion of real space navigation testing are mandatory to solve open questions around visual processing in aMCI/AD.

A recent study demonstrated that path integration performance in an immersive VR can differentiate amyloid positive from amyloid negative aMCI with high sensitivity and specificity (Howett et al., 2019). The cumulative distance error during path integration was significantly higher in amyloid positive as compared to amyloid negative aMCI patients and age-matched healthy controls. Furthermore, this behavioral effect showed a statistical correlation with entorhinal cortex volume. The entorhinal cortex is essential for path integration due to self-motion tracking by grid cell signaling (Moser et al., 2008) and known to be one of the first brain regions to be affected by tau deposition in Alzheimer’s pathology (Coughlan et al., 2018). In the aforementioned study, direct measurement of entorhinal activations during the immersive VR path integration task could not be performed due to technical limitations.

A recent real space navigation study in aMCI patients could also differentiate amyloid positive from amyloid negative aMCI patients with high diagnostic accuracy (Schöberl et al., 2020). The main difference between these two groups was an inferior performance for allocentric and egocentric route learning in amyloid positive aMCI patients as compared to amyloid negative patients, who had impaired navigation abilities only on allocentric routes (Schöberl et al., 2020). aMCI patients had decreased activations in the hippocampus, RSC, PPC, and the PFC, as the underlying correlate of a decreased spatial navigation performance. Differences in performance between both aMCI subgroups were reflected by reduced activations in the hippocampus and PPC in amyloid positive aMCI patients. aMCI patients used more horizontal search saccades and fixations compared to controls (Schöberl et al., 2020), which implies either an increased reliance on visual cues, a disturbed strategy to select relevant visual cues and incorporate them correctly into a spatial cognitive map, or a combination of both (Uiga et al., 2015). Detailed analyses of visual explorations revealed that amyloid positive aMCI patients used fewer landmarks than amyloid negative aMCI patients did. Taken together, the results from VR and real space studies suggest great potential for navigation testing for early and specific detection of aMCI.

## Navigation Testing in VR and Real Space—A Critical Discussion

**Figure 5:**
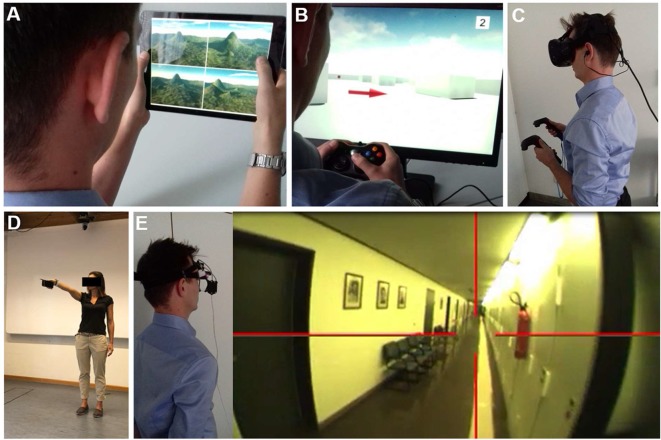
Navigation testing in virtual reality (VR) and real space. Examples of VR set-ups to study spatial navigation using **(A)** a 2D tablet touch screen paradigm (4 Mountains Test, which assesses allocentric spatial memory by altering the viewpoint, colors, and textures of the topographical layout of four mountains within a computer-generated landscape). **(B)** A 2D desktop screen paradigm with joystick navigation. **(C)** A VR paradigm displayed with a head-mounted device and motion recording. The degree of immersion increased from **(A–C)**. Functional MRI (fMRI) based studies are mostly possible in 2D paradigms and hard to achieve in immersive VR set-ups. Examples of real space spatial orientation and navigation testing by **(D)** pointing experiments to a known position in vista space (Flanagin et al., 2019) and **(E)** navigation in large-scale environments. Navigation strategy and visual exploration patterns are recorded by a head-mounted eye-tracking system and are analyzed *post hoc* in navigograms. Brain activations during real space navigation can be captured by an [^18^F]FDG-PET based approach.

Previous spatial navigation literature in humans shows a predominance of VR based on real space navigation studies (Table 1). VR set-ups combined with fMRI has given important insights into cerebral networks underlying navigation control (Maguire et al., 1998; Grön et al., 2000). The current concepts on navigation strategies like stimulus-response, egocentric or location-based, landmark-based, allocentric or spatial map based resulted mostly from studies using VR settings (Ekstrom et al., 2014; Epstein and Vass, 2014; Ekstrom and Isham, 2017; Epstein et al., 2017; Lester et al., 2017). At the beginning of VR development, VR environments were applied in a reductionistic way to study specific aspects of human navigation (i.e., processing of optic flow, perception of visual scenes, identification, selection and use of landmarks). The traditional VR set-ups (like the human variant of the Morris water maze task) were displayed on 2D screens combined with joystick navigation in a sitting position (Figures 5A,B). Novel VR technologies allow for more realistic presentations like large-scale projection with eye-tracking, and combination with physical movement on treadmill or by direct immersion of walking, eye and head movements using head-mounted VR systems (Diersch and Wolbers, 2019; Figures 5C,D). However, the currently available VR set-ups do have considerable limitations, especially if it comes to application in older subjects and patients with sensory or cognitive disorders: (1) desktop VR has a strong bias towards optic flow and visual processing and therefore does not resemble the multisensory inputs during naturalistic real space navigation; (2) treadmill walking in large-scale VR and highly immersive head-mounted VR systems allow for some degree of optic flow, vestibular and proprioceptive input. However, the dominance of optic flow and visual processing persists. Both approaches may induce subtle sensory temporal mismatch and thus lead to cybersickness (Taube et al., 2013; Diersch and Wolbers, 2019); (3) previous studies have shown that gaming experience and the degree of immersion have an impact on performance in VR tasks (Richardson and Collaer, 2011; Ruddle et al., 2011). Application of immersive VR set-ups may be especially challenging in older subjects, because of missing gaming experience and problems in immersion due to susceptibility to cybersickness. More training sessions, simpler set-ups, reduced optic flow and movement speed can improve comfort of subjects in VR (Diersch and Wolbers, 2019); and (4) immersive head-mounted VR systems cannot easily be combined with synchronous fMRI to depict navigation-induced brain activations (Diersch and Wolbers, 2019; Howett et al., 2019). Alternative approaches like *post hoc* imagination and memorization in fMRI or fMRI adaptation may be used to overcome this problem partially (Hassabis et al., 2009; Doeller et al., 2010; Morgan et al., 2011; Schinazi et al., 2013; Epstein et al., 2017). Imagination techniques in fMRI may also be used after learning spatial layouts of real environments (Howard et al., 2014; Javadi et al., 2017; Brunec et al., 2018; Patai et al., 2019). Indeed, these approaches increased our understanding of the different roles of the hippocampus, entorhinal, retrosplenial and PFC for human spatial navigation. Nevertheless, certain limitations exist, especially the lack of vestibular and somatosensory inputs during the fMRI-trial sessions. The very extensive and strict training program, which is necessary for a successful *post hoc* trial period, is somehow artificial, as the spatial layout of our surrounding environments is not learned as systematically in everyday life. While the task design can be applied successfully to young and healthy students, older subjects and patients with neurological disorders (i.e., cognitive decline, acute brain lesions) might have more problems in learning these paradigms, which might cause high dropout rates.

**Table 1:** Comparison of different virtual reality (VR) and real space-based navigation paradigms.

	Advantages	Limitations	Future applications
2D desktop	High level of control and standardization, combination with fMRI experiments possible	Overly dependency on visual processing, no vestibular or proprioceptive feedback or motor-efference signals, low degree of immersion to VR	Investigation of specific aspects of navigation control (in fMRI), quantification of spatial orientation deficits in patients with cognitive disorders or mobility restrictions
Large-screen	Analysis of eye movements and visual exploration patterns in more naturalistic and ecologically valid environments	High visual dependency, no multisensory feedback or motor-efference signals, the potential for cybersickness, hardly adaptable to online fMRI	Analysis of visual exploration strategies in environments of different scales, textures, and abundance of landmarks
Hybrid	Allows some degree of multisensory feedback and motor-efference control	Increasing risk of cybersickness by sensory mismatch, moderate immersion to VR, restricted degree of interaction, combined with online fMRI not possible	Training of spatial navigation abilities for patients with cognitive disorders or acute/subacute cerebral lesions (e.g., during in-patient rehabilitation)
Head-mounted	Highly interactive allows for multisensory inputs and motor-efference signals, multiple options to track behavior, possibility to go beyond reality	Higher risk of cybersickness, problems with embodiment (in older subjects), the dependence of performance on previous VR experience, combined with online fMRI restricted	Investigation of behavioral responses to controlled environmental changes, the potential for combination with advanced fMRI techniques or PET-based approaches
Real space navigation	Investigation of multisensory contribution to navigation control, analysis of visual exploration patterns in natural environments, navigation in 3D environments, combination with PET imaging to depict navigation-induced brain activations, no cybersickness	Problems with standardization of the navigation task, limitations in experimental manipulation of the task, problems if mobility restrictions exist, potential ceiling effects for repetitive testing	Application as an easy screening test for patients with navigation disorders in clinical routine, analysis of eye movements as a potential biomarker for navigation control, transfer to everyday life situations (by combination with mobile tracking technologies like GPS)

Despite considerable advances in the development of VR technologies and novel “hybrid-approaches” (i.e., immersive VR plus treadmill-locomotion, *post hoc* mental navigation of previously learned real space environments), human real space navigation still remains the benchmark to study the processing and integration of multiple sensory inputs during navigation in an everyday life scenario. This does not only include optic flow, vestibular and proprioceptive inputs but potentially also auditory and olfactory stimuli. All these sensory modalities have to be continuously weighted in a natural environment to extract the ones important for spatial navigation. Thus, not only the integration but also intentional suppression of rather disturbing or conflicting sensory inputs might be of high relevance during real space navigation. Real space orientation can be implemented in paradigms like pointing to known landmarks or navigating in large-scale environments (Figure 5E). Older subjects or patients with neurological disorders tend to accept and tolerate real space navigation better because they are used to it. A disadvantage of real space paradigms is the more difficult standardization and experimental manipulation. Repetitive testing in the known spatial environment may lead to a ceiling effect of navigation performance.

## Conclusions

VR and real space navigation paradigms have contributed to the understanding of human spatial navigation control and thus seem to be complementary. VR settings may have advantages for the investigation of specific aspects of navigation control, because they can be ecologically applied, excellently controlled and manipulated. Immersive VR tasks, for example, have helped to disentangle the role of the hippocampus for coding spatial and temporal metrics in a concrete manner (Hassabis et al., 2009; Morgan et al., 2011; Schinazi et al., 2013). However, VR based approaches may be limited by the over-reliance on visual input while neglecting vestibular, somatosensory and motor-efference signal processing. More recent “hybrid-approaches” such as combining head-mounted VR techniques with treadmill-locomotion or fMRI with mentalization of recently learned real environments significantly increased our understanding of the exact contribution of core components of the human spatial navigation network and the cue-dependence of spatial memory retrieval (Howard et al., 2014; Javadi et al., 2017; Huffman and Ekstrom, 2019; Patai et al., 2019). Nevertheless, real space navigation experiments with a dedicated analysis of eye movement data and navigation strategies may help to study the sensory contribution to navigation in more detail. Recent studies have clearly shown that this approach is feasible to investigate the physiological control of navigation in healthy subjects, as well as the pathology of spatial navigation disorders in patients with sensory disorders, acute cerebral lesions and chronic brain dysfunction due to neurodegeneration. It thereby can help to identify patients at risk for impending dementia. Real space navigation paradigms are potentially easier to apply to neurological patients because problems like cybersickness due to sensory mismatch can be avoided. Future strategies of navigation testing should aim to bridge the gap between laboratory and bedside conditions. Navigation testing in clinical routine needs to be time-economical and reliable in the detection of deficits of cerebral navigation control.

## Author Contributions

FS: drafting and revising the manuscript, review of literature, concept and design of article. AZ: drafting and revising the manuscript, review of literature, concept and design of article. TB: drafting and revising the manuscript, concept and design of article.

## Conflict of Interest

The authors declare that the research was conducted in the absence of any commercial or financial relationships that could be construed as a potential conflict of interest.
